# Perception and Acceptance of Shade Variances in the Smile

**DOI:** 10.3390/dj12080260

**Published:** 2024-08-16

**Authors:** Jana Wesselbaum, Dieter Dirksen, Christoph Runte, Alexander Becker

**Affiliations:** 1 Dental Practice, D-45657 Recklinghausen, Germany; 2 Department of Prosthetic Dentistry and Biomaterials, University of Muenster, D-48149 Münster, Germany; dirksdi@uni-muenster.de (D.D.); christoph.runte@ukmuenster.de (C.R.)

**Keywords:** denture teeth shade, color perception, color acceptance

## Abstract

(1) This study aimed to evaluate the influences of differences in denture teeth shade on harmony ratings and esthetic quality. Particular attention was paid to the question of how the overall variance of shade differences in the dental arch influences the perception of shade differences between adjacent teeth. (2) A total of 51 pictures of upper front teeth with standardized different colors of the left central incisor and different color variance of the dental arch were created. Eighty participants without dental knowledge and of different age, sex and educational level rated the pictures on a visual analogue scale from 0 (strongly disagree) to 100 (strongly agree) with regards to esthetic and color equality. (3) Results: The color differences between two teeth were judged in a negative linear correlation to the evaluation of color and esthetic quality. There was a sex- and education-specific difference in color and harmony ratings. In relation to the age of the participants, there was a significant difference in the color rating (*p* = 0.01) but not in the harmony rating (*p* = 0.27). Lower rating of color difference and harmony was found for a color difference in the dental arch up to ΔE = 3.1. In contrast, higher color differences resulted in higher ratings. (4) There is an influence of personal parameters on color perception. Color inhomogeneity in a dental arch leads to significant differences in color and harmony rating in a non-linear relation.

## 1. Introduction

Functionality and esthetics of teeth are important parameters for health, well-being and social integrity. Perception of shade is an essential factor for a satisfactory appearance [1]. The acceptability thresholds for shade differences in dentures were investigated for the first time in 1987 [2]. The authors examined the color stability of composites under the influence of light and the acceptability of the induced shade differences. A shade difference was rated unacceptable at a value of ΔE = 3.3.

Another study examined the perceptibility and acceptability thresholds for single implant restorations and oral mucosa evaluated by dentists, dental technicians and non-specialists. The perceptibility threshold was detected at ΔE = 2.7 (dental technicians), ΔE = 3.3 (dentists) and ΔE = 4.4 (non-specialists). The acceptability threshold was observed at ΔE = 4.4 (dental technicians), ΔE < 3.7 (dentists) and ΔE > 4.5 (non-specialists) [3]. In contrast, a similar study investigated the visual threshold using pictures with two simulated central incisors having different colors. They found a perceptibility threshold of 0.72 and an acceptability threshold of 2.62 based on the CIELAB whiteness index [4]. In 2016, a study examined thresholds for shade differences using standardized presentations of five clinical cases, which showed upper incisors and oral mucosa. They determined perceptibility thresholds at ΔE = 1.8 (dental technicians), ΔE = 1.9 (dentists) and ΔE = 1.8 (non-specialists) [5]. Similar results were obtained in 2018 [6]. The authors examined a 50:50 perceptibility threshold of ΔE = 1.9 and acceptability threshold of ΔE = 4.2. The 50:50 tolerance point is defined as the amount of shade difference at which half of participants realize a shade difference between the observed teeth. Moreover, the 50:50 tolerance point describes an equal probability of accepting or rejecting the prosthetic restoration because of shade differences [7].

A negative correlation between increasing shade difference and the ratings of color homogeneity and esthetic quality was reported [7,8]. However, harmonious shades do not lead to the highest ratings [9]. 

In addition to the shade, there are more aspects, such as gloss, reflection and transparency, that have an influence on perception. For example, the translucency acceptability threshold (TAT) was detected at ΔTAT = 4.43 and the translucency perceptibility threshold (TPT) at ΔTPT = 1.3 [10]. 

Another aspect that appears to influence the acceptability threshold is chromaticity. Observers show higher acceptability for more achromatic samples than for more chromatic samples [11].

Moreover, individual participant parameters affect color perception. On the one hand, an influence of gender [12] and, on the other hand, an influence of level of education [13] was found in previous studies. However, there is no consensus on which gender performs better in color differentiation [14,15]. With higher level of education, higher satisfaction with the appearance and esthetics of one’s own teeth was found [13]. An influence of the age of the participants was not reported in previous studies [16,17].

Furthermore, the influence of the total color variance in a dental arch on color perception is still unexplored.

Therefore, the main objective of this study was to investigate how the perception and acceptance of two neighboring teeth having different shades is influenced by shade differences in the whole dental arch. The research hypotheses tested in this study were as follows: (1) the perception and acceptance of two neighboring teeth with different shades would be influenced by shade differences in the whole dental arch. (2) The age of the participant would have an effect on color perception and acceptance. (3) The gender of the participant would have an effect on color perception and acceptance. (4) The education level of the participant would have an effect on color perception and acceptance.

## 2. Materials and Methods

### 2.1. Dental Arch Models

**Figure 1:**
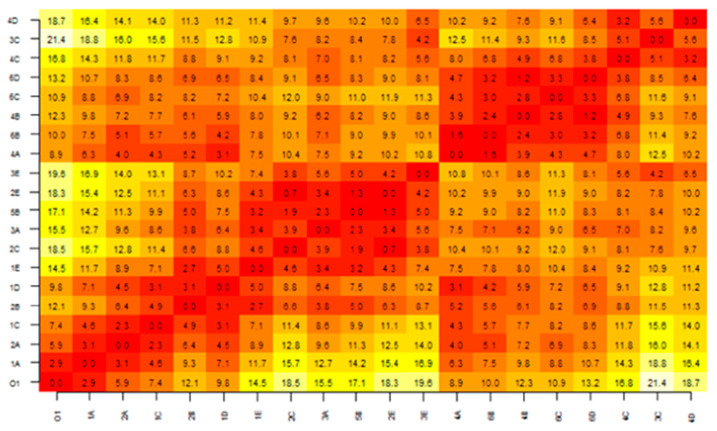
Distance matrix containing ΔE values of measured denture teeth.

**Figure 2:**
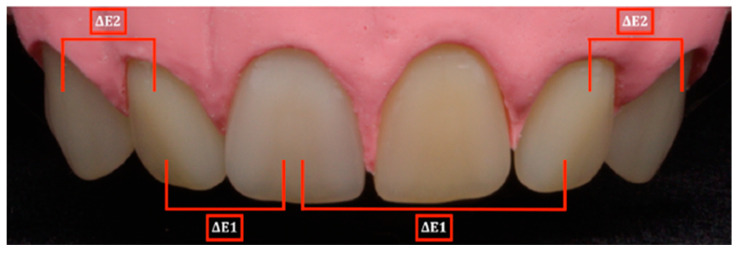
Dental arch model with designations of the teeth used for the color distance calculations (ΔE1 = color distance of reference tooth and lateral incisor, ΔE2 = color distance of canine and lateral incisor).

For investigation of the influence of color differences in a dental arch on color and harmony perception, participants had to rate photographs of dental arch models having denture teeth with different shades. In preparation for the selection of suitable laboratory color values and color differences, an instrumental color measurement was carried out on twenty denture teeth (Vivodent S PE, Ivoclar Vivadent AG, Ellwangen, Germany) having different shades (O1, 1A, 2A, 1C, 2B, 1D, 1E, 2C, 3A, 5B, 2E, 3E, 4A, 6B, 4B, 6C, 6D, 4C, 3C, 4D) using a spectrophotometer (Shadepilot, Degudent, Hanau, Germany). Color differences were represented as CIELab color distances (ΔE values) in a distance matrix (Figure 1). Those teeth were considered that corresponded to the variety of shade distances occurring in the natural dental arch. According to the distance matrix, the most possible combinations resulted when using tooth shade 6B, 4A or 4B as the reference tooth. These three shades have relatively small and preferably linear shade distances in a range from ΔE = 1 to about ΔE = 12 to one of the other denture teeth. The right first incisor (11) of shade 4A serves as the reference tooth in this study, as it best met the requirements described above. Ten neighboring incisors (tooth 21) were selected, whose perceptible shade distances to the reference tooth had ΔE values of 1.6 to 12.5. In addition, a variation of perceptible shades for teeth 13, 12, 22 and 23 were chosen. Henceforth, these are described as the color/shade difference of the dental arch. These teeth were assembled with similar color distance to that of the canine and lateral incisor (ΔE2) and similar color distance to that of the reference tooth and lateral incisor (ΔE1) (Figure 2). The esthetic features of the natural dental arch were taken into account, so that the canines were darker than the incisors. Five versions of the dental arch were constructed (Table 1). The first version contained the same shade as the reference tooth. Versions 2 to 5 were characterized by increasing color distances. The left upper central incisor was changed ten times in each version. This resulted in 50 different dental arches (Table 2 and Table 3), which were assessed by all the participants. Gingiva was imitated using silicone (Implant Mask, Henry Schein, Langen, Germany).

### 2.2. Photograph Recording and Editing

Each dental arch was homogeneously illuminated with daylight fluorescent tubes on a black background in a light box. Pictures were taken with a Nikon D7000 camera (lens: Nikkor 16–85 mm, 1:3.5–5.6; Nikon, Minato, Japan; aperture: f/20) mounted on a tripod. It was visually checked that the dental arch was consistently in focus over the entire area. For color calibration, the ColorChecker Passport (Classic Target, X-Rite, Grand Rapids, MI, USA) was used. For this purpose, a reference image of a color chart containing 24 color patches provided by the calibration system was captured and a DNG profile was calculated using the ColorChecker software v1.1.1. This profile was subsequently assigned to the images that were taken under the same illumination conditions. Pictures were aligned and trimmed using the software RawTherapee (Version 5.7, Gábor Horváth and RawTherapee development team, open-source software) to a size of 1259 × 414 pixel (Figure 2).

### 2.3. Image Presentation

**Figure 3:**
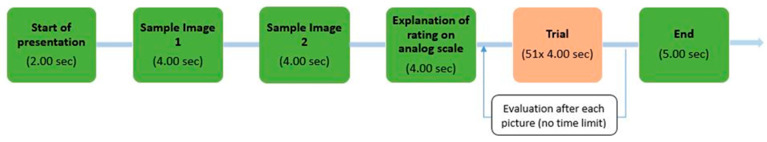
Flow chart of presentation.

The images of the dental arches were presented on a MacBook Pro (Retina, 13”, 2012, Apple, Cupertino, CA, USA), which was color calibrated using i1 Display Pro and i1 Profiler (X-Rite, Grand Rapids, MI, USA). A standardized procedure was programmed using PsychoPy3 (Jonathan Peirce and Open Science Tools Ltd., Nottingham, UK), a toolbox for flow control of image presentations and ratings based on the language Python. First, two examples were shown, which were not included in the assessment, to familiarize the subjects with the procedure. Assessment was started with an explanation of how to rate a shown picture by clicking on the visual analogue scale. The ratings on the visual analogue scale were converted internally into a numerical range from 0 to 100 for evaluation. Subsequently, each picture was shown for four seconds. After each picture, the participants were either asked to rate whether the right and left central incisors had the same color or to rate whether the combination of shades was harmonious. The first question was asked for detecting evaluation of color and the second for detecting evaluation of harmony. The participants were asked to rate the statement on a visual analogue scale from “strongly agree” to “strongly disagree” by clicking on the scale (the original study questions and visual analogue scale being in German). Rating was not time limited. After the first assessment rating, the same picture was shown again and the second statement was inserted. Without consideration of rating time, the trial lasted approximately seven minutes. The sequence of images was different for each participant and was randomly selected by the program. The results were stored in a pseudonymized form. In Figure 3, the flow chart of presentation is shown.

### 2.4. Participants

Eighty adult subjects with no specific prior knowledge of color assessment participated in the study. Inclusion criteria were a completed school education and normal color vision. The latter was ensured via an Ishihara color test using plates 1, 2, 7, 8, 11 and 14. Participation was voluntary, and no incentives were offered. The participants were briefed about the course of the experiment and data protection. Written declaration of consent was made. The study was approved by the Ethics Committee of the Medical Faculty of the University of Münster (2015-630-f-S).

### 2.5. Statistical Methods

Pseudonymized results were saved in Excel2020 (Microsoft, Redmond, Washington, DC, USA). Statistical analyses were performed using R (Version 3.3.3, R Core Team) and RStudio (RStudio PBC, Boston, MA, USA). Linear regression analyses were performed to determine the influence of the independent variables (ΔE values, age and color difference) on color homogeneity and harmony, which represent the dependent variables. Normal distribution was tested using the Shapiro–Wilk test. The Wilcoxon and Kruskal–Wallis tests were used for analyzing non-normally distributed data, followed by the Nemenyi test (post hoc test) for detecting significant differences between the groups. The level of significance was set at α = 0.05.

In addition, the perceptibility and acceptability thresholds were examined using the CIELab and CIEDE2000 color difference formulas, because the CIEDE2000 formula better reflects the color differences perceived by the human eye [18]. Therefore, the ΔE00 values were additionally calculated using the implementation proposed by a study from 2005 [19].

### 2.6. Data Availability

All data from this study are presented within Section 3 or are available from the corresponding authors upon reasonable request.

## 3. Results

### 3.1. Participants

Eighty participants took part in the study. A total of 36 were female (gender group 1) and 44 male (gender group 2). The age range was 18 to 76. Accordingly, participants were divided into four age groups (age group 1: teenagers, age 18–25; age group 2: young adults, age 26–40; age group 3: adults, age 41–59; age group 4: older people, age 60–76). Distribution of the participants into age groups is shown in Table 4. Moreover, the participants were divided into two education groups (education group 1: low education level, main school degree or intermediate educational qualification; education group 2: high education level, general high school diploma or technical college entrance qualification). The distribution is also shown in Table 4.

### 3.2. Evaluation of Color and Harmony

#### 3.2.1. Frequency Distribution of Ratings

**Figure 4:**
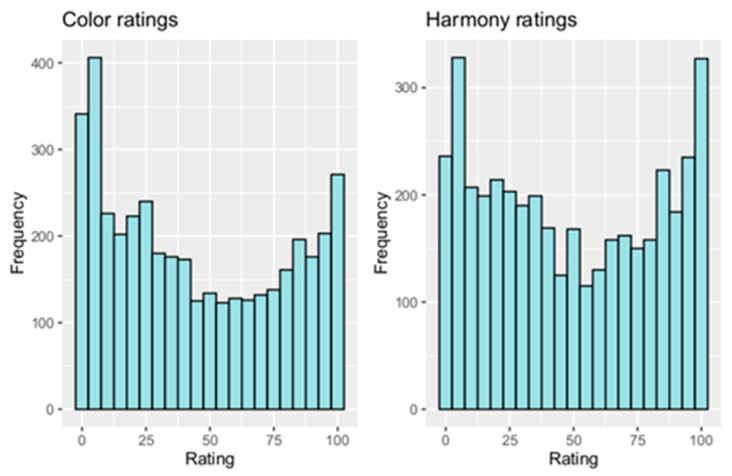
Cumulative frequency distribution of color and harmony ratings (y-axis = frequency, x-axis = rating on visual analogue scale).

Cumulative frequency distributions of color and harmony are shown in Figure 4. The evaluation resulted in 4080 ratings. A large spread of ratings was detected. Many participants opted for extreme values in their evaluation of color and harmony. Most ratings were made in the value ranges of 0–25 and 85–100.

#### 3.2.2. Influence of Color Distance (ΔE Value) on Ratings

To investigate the influence of color distance on ratings, the averages of the ratings for the shown pictures were examined. Averaged evaluations of color and harmony as functions of the shade differences of upper central incisors (ΔE values 0.0 to 12.0) are shown in Table 5. Rating was between 14.58 and 75.75 for color equality and between 17.65 and 78.50 for harmony in the dental arch. The harmony was rated higher on the visual analogue scale than was the color in general. It could be noticed that pictures without shade differences received lower color ratings than the pictures having color distances of ΔE = 1.6 and ΔE = 3.1.

**Figure 5:**
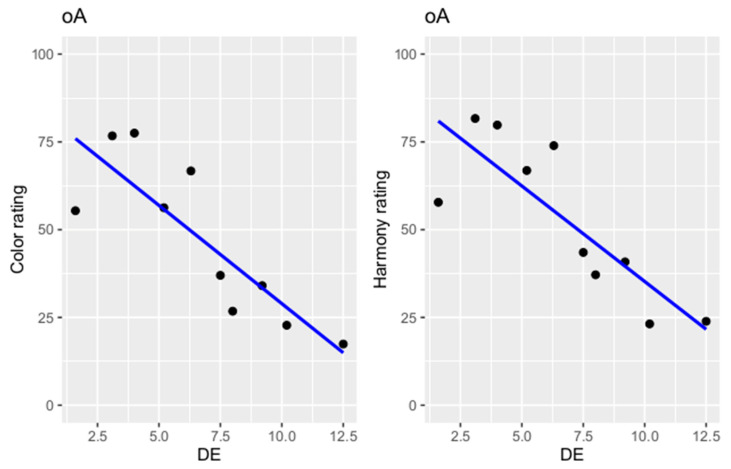
Scatter plot of color and harmony ratings (regression line in blue; oA = participant ratings, in which teeth 13, 12, 22 and 23 had shade 4A and there was no color variance in the dental arch; DE = ΔE value).

There was a negative relationship between the ΔE value and the ratings of the participants. Increasing ΔE values were rated less color-matched and increasingly inharmonious (Figure 5). Increasing ΔE value by one unit resulted in a decrease of the color score by 6.50 points and of the harmony by 6.40 points (Table 6). A significant difference between the ratings of color and harmony and the ΔE value was detected in a regression analysis (*p* < 0.0001).

#### 3.2.3. Influence of Shade Variance in the Dental Arch on Ratings

**Figure 6:**
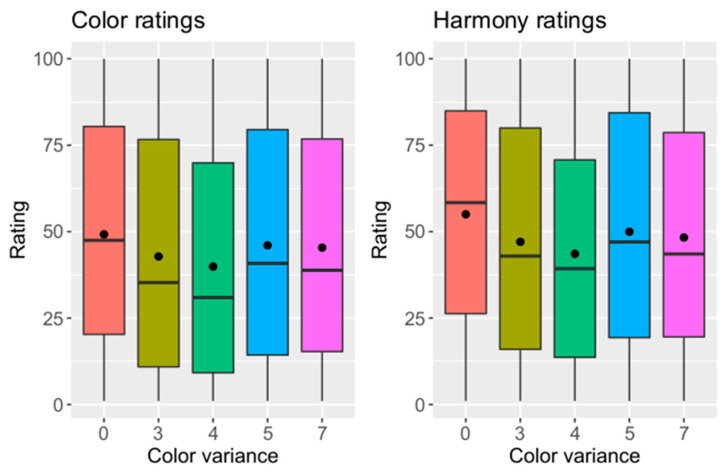
Color and harmony ratings influenced by color variance in dental arch (y-axis = rating on visual analogue scale, x-axis = code of color variance (VarEK); 0 = ΔE1 = 0, ΔE2 = 0; 3 = ΔE1 = 3.1, ΔE2 = 3.1; 4 = ΔE1 = 4.0, ΔE2 = 4.5; 5 = ΔE1 = 5.0, ΔE2 = 5.2; 7 = ΔE1 = 7.5, ΔE2 = 7.0).

To evaluate the influence of shade difference in the dental arch, shade variance was coded as shown in Table 7. The relationship is shown in Figure 6. The averaged ratings decreased with increasing color inhomogeneity until ΔE1 = 4.0 and ΔE2 = 4.5. A Kruskal–Wallis test was used to evaluate differences between shade variances. A significant difference was detected (*p* < 0.001). A Nemenyi post hoc test showed significant differences in color rating between VarEK 3 and 0, VarEK 4 and 0, VarEK 5 and 4 and VarEK 7 and 4 (Table 8). A corresponding test revealed significant differences in harmony rating between VarEK 3 and 0, VarEK 4 and 0, VarEK 5 and 0, VarEK 7 and 0, VarEK 5 and 4 and VarEK 7 and 4. The color and harmony ratings showed a negative relation to the ΔE values of the left upper central incisor. A linear dependence of the shade differences in the dental arch on the ratings was not observed, as demonstrated by a regression analysis (color rating: *p* = 0.56, harmony rating: *p* = 0.35).

#### 3.2.4. Influence of Age of Participants on Ratings

**Figure 7:**
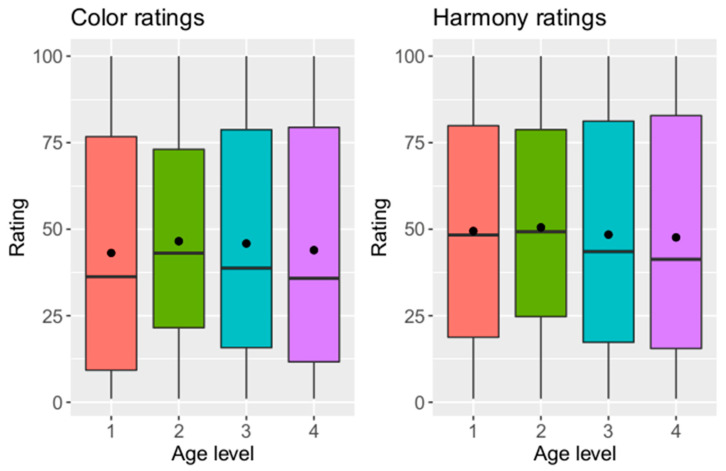
Color and harmony ratings depending on age of participants (y-axis = rating on visual analogue scale, x-axis = age level; 1 = 18–25, 2 = 26–40, 3 = 41–59, 4 = 60–76).

Depending on the age of the participants, the color and harmony ratings are shown in Figure 7. Only small differences of the distributions between the age groups can be observed. Shapiro–Wilk tests showed that normal distributions cannot be assumed (*p* < 0.05). Kruskal–Wallis tests detected significant differences between the age groups in color rating (*p* = 0.01) and no significant differences in harmony rating (*p* = 0.27). A Nemenyi post hoc test showed significant differences in color rating between age groups 1 and 2 (*p* = 0.014). There was no linear dependency between age and color ratings in a regression analysis (*p* = 0.69), but there was a linear dependency between age and harmony rating (*p* = 0.043). However, explanatory potential was low (coefficient of determination R2 = 0.11).

#### 3.2.5. Influence of Gender of Participants on Ratings

**Figure 8:**
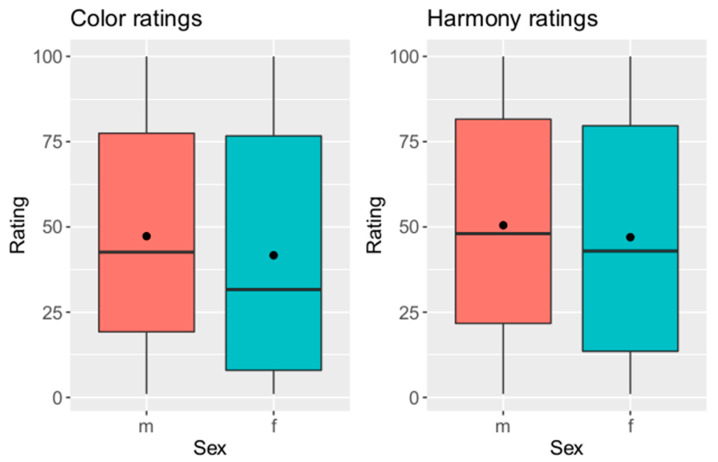
Color and harmony rating depending on sex of participants (y-axis = rating on visual analogue scale, x-axis = sex; m = male, f = female).

Figure 8 shows the color and harmony ratings depending on gender. A Wilcoxon test showed significant differences in color and harmony rating between women and men (color rating: *p* < 0.001, harmony rating: *p* < 0.001). Women gave lower ratings in color and harmony rating than men. Moreover, the spread of the ratings of female participants was larger. Harmony rating was higher than color rating in both sex in general. There was a negative relation between ratings and ΔE values of changed left upper central incisor.

#### 3.2.6. Influence of Education Level of Participants on Evaluation

**Figure 9:**
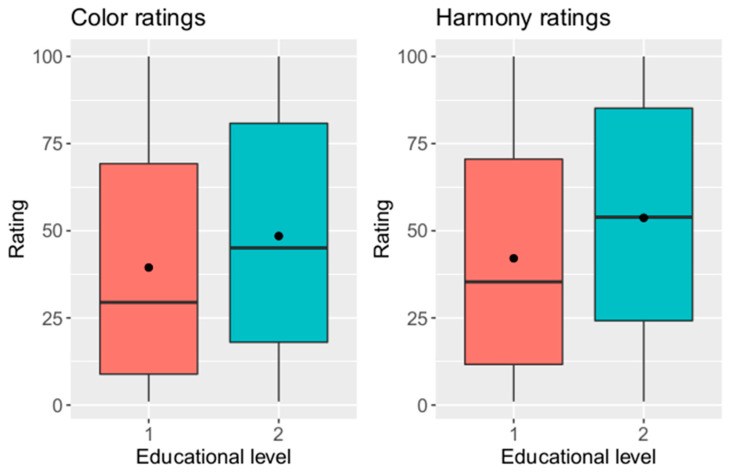
Color and harmony ratings depending on education level of participants (y-axis = rating on visual analogue scale, x-axis = educational level; 1 = main school degree or intermediate educational qualification, 2 = general high school diploma or technical college entrance qualification).

Depending on age of participants, color and harmony ratings are shown in Figure 9. A Wilcoxon test revealed a significant difference in color and harmony ratings between the two groups (*p* < 0.001). Participants having higher education level (education group 2) rated color and harmony higher than participants having lower education level (education group 1). In addition, there was a negative dependency of rating and shade difference. However, this dependency seems to be higher in the group having higher education level.

### 3.3. Perceptibility and Acceptability Thresholds Using CIELAB Color Difference Formula

**Figure 10:**
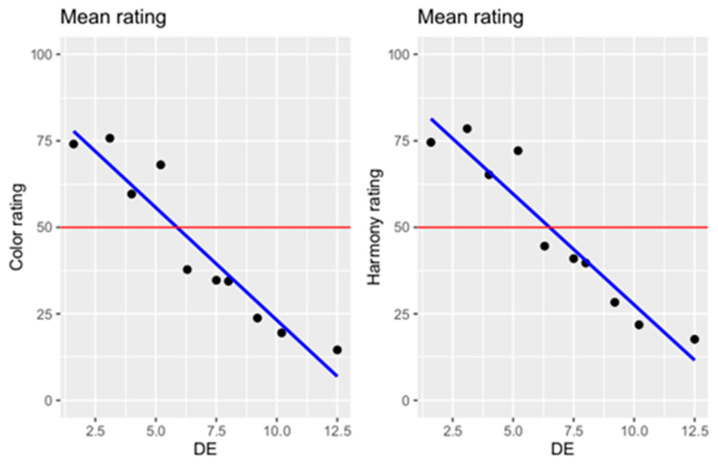
Perceptibility threshold (**left**) and acceptability threshold (**right**) (y-axis = rating on visual analogue scale, x-axis = ΔE value).

Here, the perceptibility threshold is defined as ΔE = 5.87, while the acceptability threshold is defined as ΔE = 6.50. The thresholds are shown in Figure 10.

### 3.4. Perceptibility and Acceptability Thresholds Using CIEDE2000 Formula

In addition, the perceptibility threshold using the CIEDE2000 color difference formula was detected at ΔE00 = 3.69 and the acceptability threshold at ΔE00 = 4.16. Table 9 shows the color distances of shades using the CIEDE2000 formula in relation to the CIELab formula.

## 4. Discussion

### 4.1. Color and Harmony Rating

The current study revealed a negative correlation of the ratings of color homogeneity and esthetic quality to increasing shade difference. This is in accordance with the findings of previous studies [7,8]. The higher perceptibility and acceptability thresholds could be due to the use of an in vivo study design. Previous investigations detected higher thresholds in in vivo studies [1,13]. The color and harmony rating of the picture without shade difference between the right and left upper central incisor (ΔE = 0) was striking. A high rating of nearly 100 on the analogue scale was expected, but an average of 70.83 (color rating) and 76.68 (harmony rating) was observed. Similar results were found in 2007 [9], although a reason could not be determined.

### 4.2. Influence of Overall Shade Difference and Color Inhomogenity

A significant influence of shade difference and color inhomogeneity in the dental arch on color and harmony rating was found. Increasing shade difference to ΔE = 3.1 resulted in lower ratings for color equality of the central incisor and esthetic quality of the dental arch. Increasing color inhomogeneity in the dental arch seems to be noticed as inharmonious. In addition, color differences are perceived more strongly. On the other hand, higher shade differences (ΔE = 5.0, ΔE = 7.0) were rated more highly. There seems to be no linear correlation between higher overall shade variation in the dental arch and lower color and harmony rating. Shade difference seems to have negative influence on color and harmony rating until a specific ΔE value. Above this value, it appears to have the reverse effect. From a certain heterogeneity of the tooth shade in the dental arch, the perceived shade difference between two adjacent teeth is significantly smaller and is perceived as less unesthetic. Comparable studies are currently lacking. However, it could be suggested that there is an influence of shade difference in the dental arch on color perception and acceptance in general. As a current unique selling proposition of this study, it cannot be resolved whether this influence leads to higher perception of shade difference and the dental arch being rated as less harmonious. Observations are limited by the rough gradation of color differences in the dental arch and the consequent inaccurate definition of a turning point at which shade difference has a positive or negative influence. Knowledge of this effect could help in restoration for patients with removable dentures, where shade in the dental arch is differing, or where existing prosthetic restorations, for example crowns and bridges, do not have an equal color. In these patients, the choice of tooth shade may play a lesser role in the subsequent subjective assessment of esthetics than previously assumed. To substantiate these results, further research is needed.

### 4.3. Influence of Age on Rating

A weak influence of age on harmony rating was observed in the current study. However, the effect has only minor relevance. No increasing change in the ability to judge shade difference could be observed with increasing age. In comparison to a similar study, which examined color perception using the Farnsworth–Munsell 100 color test and could not detect an influence of age [17], this result seems to be realistic. However, further studies are needed to assess the impact of age on color perception and harmony rating.

### 4.4. Influence of Gender on Rating

The current study found a significant difference of color perception and rating between women and men. Men’s ratings were higher than women’s. Sex-specific differences in color vision based on genetics are conceivable. Two types of color receptors are encoded on the X chromosome. Therefore, men, having only one X chromosome, are affected by color vision deficiency (CVD) more often than women. The influence of color blindness was excluded by performing a color vision test on the participants before rating. However, individual differences in distribution and ratio of color receptors on the retina can lead to small differences between women and men [12]. In addition, similar results were detected in various studies, although there is no consensus which gender has better color differentiation [14,15].

### 4.5. Influence of Education Level on Rating

There was a significant influence of education level on color and harmony rating. The participants with higher education gave higher ratings than the participants with lower education. The influence of education level on color perception has not been investigated in previous studies. Level of education was only considered in studies that investigated the participants’ satisfaction with the appearance of their own teeth and the esthetics of the upper incisors [13]. They found higher satisfaction occurring with higher educational level.

It can be assumed that color differences are rated less critically or that prosthetic restoration was better when participants had higher educational levels. Therefore, further research is needed.

### 4.6. Influence of the Use of Different Color Difference Formulas

The use of different metrics for the calculation of color distances in the CIELab color space leads to deviating results. While the Euclidean distance measure is the most widely used in studies and for this reason was used here, there is a whole range of alternative measures that promise to describe the perceived color distances more accurately [18,19,20]. One of these is CIEDE2000, which was therefore employed here for comparison. This resulted in significantly lower perceptibility and acceptability thresholds, but it did not change the general results and conclusions.

### 4.7. Limitations of the Study

The selected tooth shades and shade differences represent a limitation of the study. The deliberate selection of visibly different shades allowed the evaluation of the subjects’ assessments of the shade differences and the resulting esthetic perceptions. However, the selected color differences could have distorted the threshold value for the perception of color difference. 

In addition, the use of denture teeth instead of real teeth could represent a limitation in the assessment of color differences. However, since the use of denture teeth made it possible to standardize the color differences—which, in the view of the authors, provided better interpretation of the results—it was decided to use the test setup described. 

Furthermore, natural teeth, denture teeth and restorative materials can change color over time due to external influences such as liquids and smoke [21]. This could not be taken into account in the present study. However, in the case of an age-related color change, this is likely to occur in a generalized manner, so that the perception and acceptance of the color differences should remain at a similar level. This hypothesis could provide an approach for further investigation. 

Moreover, it is possible that the participants improved their performance or became tired during the rating process. The familiarization phase should have mitigated this, but differences in individual learning curves and fatigue levels could still influence the results. 

The use of a visual analogue scale for subjective ratings (color similarity and harmony) could also have led to different participants interpreting and using the scale differently. Personal preferences and previous experiences can strongly influence these subjective ratings.

Nevertheless, the present study allows conclusions to be drawn about the esthetic evaluation of two adjacent teeth having shade differences in the dental arch, which was the focus of this study. 

## 5. Conclusions

Within the limitations of this study, the following conclusions were made:

1. The color difference between two observed objects is in negative relationship to the rating of color homogeneity and esthetic quality. However, there is a nonlinear dependence of the ratings of color and harmony on the color differences not only of the directly adjacent teeth.
2. The age of the participants has a low effect on color and harmony rating. There is a statistically significant difference in color ratings between participants aged 26–40 and those aged 18–25. However, the explanatory potential is low.
3. The gender of the participants influences the rating of color homogeneity and esthetic quality. Women evaluate color homogeneity and esthetic quality more critically and give lower ratings than men.
4. The education level of the participants has an effect on rating of color homogeneity and esthetic quality. A higher education level is related to higher ratings.

A new finding was the nonlinear dependence of the ratings of color and harmony on the color differences not only of the directly adjacent teeth. This point needs to be clarified by further research using larger sample sizes and comparison of results with dentists’ assessments. A similar study is planned for this purpose, in which dentists will also participate.

## Figures and Tables

**Table 1:** Versions of dental arch model (ΔE1 = color distance of reference tooth and lateral incisor, ΔE2 = color distance of canine and lateral incisor).

Version	Reference Tooth	Second Incisor	Canine	ΔE1	ΔE2
1	4A	4A	4A	0.0	0.0
2	4A	1D	2B	3.1	3.1
3	4A	2A	1D	4.0	4.5
4	4A	2B	5B	5.0	5.2
5	4A	3A	4C	7.5	7.0

**Table 2:** Shade distribution in dental arch.

Version	Study Name	Tooth in Dental Arch	Shade
1	oA	13	4A
		12	4A
		11	4A
		21	changed
		22	4A
		23	4A
2	D3	13	2B
		12	1D
		11	4A
		21	changed
		22	1D
		23	2B
3	D4	13	1D
		12	2A
		11	4A
		21	changed
		22	1D
		23	2A
4	D5	13	5B
		12	2B
		11	4A
		21	changed
		22	2B
		23	5B
5	D7	13	6B
		12	3A
		11	4A
		21	changed
		22	4A
		23	6B

**Table 3:** Shades of changed tooth (21).

Shades of Changed Tooth (21)
4A (only in version 1)
6B
1D
2A
2B
1A
3A
4C
5B
2E
3C

**Table 4:** Distribution of participants.

Individual Parameter	Group/ Code	Definition	Quantity (n = 80)
Age	1	age between 18 and 25 (teenagers)	21
	2	age between 26 and 40 (young adults)	16
	3	age between 41 and 59 (adults)	21
	4	age between 60 and 76 (older people)	22
Gender	1	Woman	36
	2	Man	44
Education level	1	main school degree or intermediate educational qualification (low education level)	33
	2	general high school diploma or technical college entrance qualification (high education level)	47

**Table 5:** Mean color and harmony ratings.

ΔE Value	Mean Color Rating	Mean Harmony Rating
0.0	70.83	76.68
1.6	74.07	74.56
3.1	75.75	78.50
4.0	59.63	65.22
5.2	68.06	72.13
6.3	37.80	44.57
7.5	34.74	40.97
8.0	34.43	39.70
9.2	23.79	28.34
10.2	19.51	21.84
12.5	14.58	17.65

**Table 6:** Regression analysis (SD = standard deviation, T = t-value).

Rating	Coefficient	Regression Coefficient	SD	T	Significance
color rating	y-axis/y-intercept	88.19	5.63	15.67	1.74 × 10^−7^ *
	DE	−6.50	0.75	−8.65	2.493 × 10^−5^ *
harmony rating	y-axis/y-intercept	91.62	5.29	17.33	1.25 × 10^−7^ *
	DE	−6.40	0.71	−9.06	1.76 × 10^−5^ *

* *p* < 0.001.

**Table 7:** Code for shade difference in dental arch.

Dental Arch Version	Code of Shade Variance (VarEK)	ΔE1	ΔE2
1 (oA)	0	0	0
2 (D3)	3	3.1	3.1
3 (D4)	4	4.0	4.5
4 (D5)	5	5.0	5.2
5 (D7)	7	7.5	7.0

**Table 8:** Nemenyi post hoc test influence of shade difference in dental arch.

	Color Rating					Harmony Rating				
	VarEK	0	3	4	5	VarEK	0	3	4	5
shade difference	3	0.000				3	6.1 × 10^−96^			
	4	0.000	0.359			4	0.000	0.228		
	5	0.197	0.314	0.002		5	0.017	0.345	0.001	
	7	0.127	0.429	0.004	1.000	7	0.000	0.906	0.026	0.867
age group	VarEK	1	2			VarEK	1	2	3	
	2	0.014				2	0.890			
	3	0.060	0.905			3	0.870	0.480		
	4	0.640	0.212	0.531		4	0.630	0.250	0.970	

**Table 9:** Color distances of shades using the CIEDE2000 formula.

Model Version	ΔE_1 (CIELab)	ΔE_2 (CIELab)	ΔE00_1 (CIEDE2000)	ΔE00_2 (CIEDE2000)
1	0.0	0.0	0.0	0.0
2	3.1	3.1	2.2	2.0
3	4.0	4.5	3.4	3.7
4	5.0	5.2	5.2	3.0
5	7.5	7.0	3.9	5.3

## Data Availability

All data from this study are presented within Section 3 or are available from the corresponding authors upon reasonable request.
